# A Deficiency of the Psychiatric Risk Gene DLG2/PSD-93 Causes Excitatory Synaptic Deficits in the Dorsolateral Striatum

**DOI:** 10.3389/fnmol.2022.938590

**Published:** 2022-07-28

**Authors:** Taesun Yoo, Shambhu Joshi, Sanjaya Prajapati, Yi Sul Cho, Jinkyeong Kim, Pil-Hoon Park, Yong Chul Bae, Eunjoon Kim, Soo Young Kim

**Affiliations:** ^1^Center for Synaptic Brain Dysfunctions, Institute for Basic Science, Daejeon, South Korea; ^2^College of Pharmacy, Yeungnam University, Gyeongsan, South Korea; ^3^Department of Anatomy and Neurobiology, School of Dentistry, Kyungpook National University, Daegu, South Korea; ^4^Department of Biological Sciences, Korea Advanced Institute of Science and Technology, Daejeon, South Korea

**Keywords:** MAGUK, dorsolateral striatum, spiny projection neurons, corticostriatal transmission, schizophrenia risk gene

## Abstract

Genetic variations resulting in the loss of function of the discs large homologs (DLG2)/postsynaptic density protein-93 (PSD-93) gene have been implicated in the increased risk for schizophrenia, intellectual disability, and autism spectrum disorders (ASDs). Previously, we have reported that mice lacking exon 14 of the *Dlg2* gene (*Dlg2*^–/–^ mice) display autistic-like behaviors, including social deficits and increased repetitive behaviors, as well as suppressed spontaneous excitatory postsynaptic currents in the striatum. However, the neural substrate underpinning such aberrant synaptic network activity remains unclear. Here, we found that the corticostriatal synaptic transmission was significantly impaired in *Dlg2*^–/–^ mice, which did not seem attributed to defects in presynaptic releases of cortical neurons, but to the reduced number of functional synapses in the striatum, as manifested in the suppressed frequency of miniature excitatory postsynaptic currents in spiny projection neurons (SPNs). Using transmission electron microscopy, we found that both the density of postsynaptic densities and the fraction of perforated synapses were significantly decreased in the *Dlg2*^–/–^ dorsolateral striatum. The density of dendritic spines was significantly reduced in striatal SPNs, but notably, not in the cortical pyramidal neurons of *Dlg2*^–/–^ mice. Furthermore, a DLG2/PSD-93 deficiency resulted in the compensatory increases of DLG4/PSD-95 and decreases in the expression of TrkA in the striatum, but not particularly in the cortex. These results suggest that striatal dysfunction might play a role in the pathology of psychiatric disorders that are associated with a disruption of the *Dlg2* gene.

## Introduction

Discs large homologs (DLG2), also known as postsynaptic density protein-93 (PSD-93) or Chapsyn-110, is a member of the membrane-associated guanylate kinase (MAGUK) family that directly interact with receptors, including AMPA and NMDA as a postsynaptic scaffolding protein (Brenman et al., 1996; Kim et al., 1996; Sheng and Sala, 2001; Sheng and Hoogenraad, 2007). Studies using a large-scale genetic analysis of human patients have indicated that the disruption of the *Dlg2* gene is closely associated with psychiatric disorders, including schizophrenia (Walsh et al., 2008; Kirov et al., 2012; Fromer et al., 2014; Sanders et al., 2022), intellectual disability (Reggiani et al., 2017), and autism spectrum disorder (ASD) in humans (Egger et al., 2014; Ruzzo et al., 2019). Despite emerging evidence of its implication in psychiatric and neurodevelopmental disorders, it remains unclear the mechanism by which a DLG2/PSD-93 plays a pathological role.

During mid-fetal development, DLG2/PSD-93 is abundantly expressed in the human striatum (Kang et al., 2011). Structural alterations of the putamen, a part of the dorsal striatum in the human brain, are found to be linked to mutations in the *Dlg2* gene (Hibar et al., 2015). Furthermore, a recent study employing longitudinal imaging combined with genetic analyses has demonstrated that single nucleotide polymorphisms (SNPs) in DLG2/PSD-93 are associated with structural asymmetry of subcortical regions, including putamen in patients with Alzheimer’s disease (AD) (Wachinger et al., 2018). In particular, corticostriatal dysfunction has been implicated in ASDs (Peça et al., 2011; Bentea et al., 2020), obsessive-compulsive disorders (Burguiere et al., 2015), and schizophrenia (Zandbelt et al., 2011; Wagshal et al., 2014), implying that striatum-related circuitries play a critical role in the pathology. In this context, we have previously found that *Dlg2* mRNA is highly expressed in the mouse striatum during early developmental stages. Mice lacking DLG2/PSD-93 have suppressed excitatory synaptic inputs onto spiny projection neurons (SPNs)–the output cell type in the striatum, as measured by spontaneous excitatory postsynaptic currents (sEPSCs), which accompanied aberrant behaviors, including suppressed locomotor response to novelty, decreased social interaction, and increased repetitive behavior (Yoo et al., 2020), that are tightly associated with dysfunction of striatal circuitries (Welch et al., 2007; Shmelkov et al., 2010; Ishii et al., 2016; Leonzino et al., 2019; Li and Pozzo-Miller, 2020). However, the underlying neural substrate for a DLG2/PSD-93 deficiency still remains elusive.

In this study, we investigated the consequence of the genetic disruption of DLG2/PSD-93 on striatal synaptic connectivity. We found that a DLG2/PSD-93 deficiency significantly suppressed corticostriatal synaptic transmission accompanying the reduced number of functional excitatory synapses in the striatum. Spine densities were significantly reduced, as well as the expression of synapse-related molecules was altered in the dorsolateral striatum, neither of which was found in the cortex of *Dlg2*^–/–^ mice. Our findings suggest a crucial role for DLG2/PSD-93 in the regulation of striatum-related circuitries, shedding a light on the potential involvement of striatal dysfunction in the pathology of various psychiatric disorders accompanying disruption of the *Dlg*2 gene.

## Materials and Methods

### Animals

*Dlg2*^–/–^ mice carrying a deletion of exon 14 of the *Dlg2* gene were generated and maintained as previously reported (Yoo et al., 2020). Briefly, all animals were fed *ad libitum* and housed under a 12-h light/dark cycle. Pups were weaned at postnatal day 21, and 3–6 mice were grouped in each cage. Only male adult mice were used for experiments. *Dlg2*^–/–^ mice were genotyped by polymerase chain reaction (PCR) using the following primers as previously reported (Yoo et al., 2020): WT allele (312 bp), 5′-CCA GAA TGT AC TTC AGC ACC A –3′ (forward) and 5′-TCG TGGTATCGTTATGCGCC-3′ (reverse); mutant allele (527 bp), 5′-GCC AAG ACT TTT AGA GAC AGC C-3′ (forward) and 5′-AAG CAG GCA ATT CAC ACC AC-3′ (reverse). The use of mice was approved by and performed in accordance with the committees of animal research at KAIST (KA2021-070) and Yeungnam University (2020-037).

### Electrophysiology

Acute coronal brain slices for the dorsolateral striatum were obtained following the protective recovery method (Ting et al., 2014). In brief, mice at 3–5 months were anesthetized with an intraperitoneal injection of a ketamine (120 mg/kg)-xylazine (10 mg/kg) cocktail and transcardially perfused with a protective buffer (NMDG aCSF) at room temperature consisting of, in mM: 100 NMDG, 12 NAC, 30 NaHCO_3_, 20 HEPES, 25 glucose. 2 thiourea, 5 Na-ascorbate, 3 Na-pyruvate, 2.5 KCl, 1.25 NaH_2_PO_4_,0.5 CaCl_2_, and 10 MgSO_4_. Mouse brains were extracted and sectioned (300 μm for whole-cell recording, 400 μm for field recording) in NMDG aCSF buffer at room temperature bubbled with 95% O_2_ and 5% CO_2_ gases using Leica VT 1200. The slices were transferred to a 32°C holding chamber containing NMDG aCSF for 11 min, followed by recovery for over 1 h in a chamber at room temperature containing a buffer (in mM: 92 NaCl, 12 NAC, 30 NaHCO_3_, 20 HEPES, 25 Glucose. 2 Thiourea, 5 Na-Ascorbate, 3 Na-Pyruvate, 2.5 KCl, 1.25 NaH_2_PO_4_, 2.5 CaCl_2_, and 1.3 MgSO_4_) oxygenated with 95% O_2_ and 5% CO_2_ gases. During all recordings, brain slices were maintained in a submerge-type recording chamber perfused with 27.5–28.5°C aCSF (2 ml min^–1^; in mM: 124 NaCl, 25 NaHCO_3_, 10 Glucose, 2.5 KCl, 1 NaH_2_PO_4_, 2.5 CaCl_2_, and 1.3 MgSO_4_ oxygenated with 95% O_2_ and 5% CO_2_ gases). Recording glass pipettes from borosilicate glass capillaries (Harvard Apparatus) were pulled using an electrode puller (Narishige). All-electric responses were amplified and filtered at 2 kHz (Multiclamp 700B, Molecular Devices) and then digitized at 10 kHz (Digidata 1550, Molecular Devices).

For extracellular field recordings, a platinum/iridium concentric bipolar stimulation electrode (CBBRC75; inner pole diameter, 25 μm) and glass recording electrodes filled with aCSF were placed on the inner border of the corpus callosum, containing mainly passing cortical axons, and the dorsolateral striatum, approximately 400 μm away from the stimulus, respectively. Paired pulse ratio was measured by applying three paired pulses at different inter-stimulus intervals (25, 50, 75, 100, 200, and 300 ms), averaging across triplicate measurements. Stable baseline responses were monitored for 5 min before the recording. For input-output curves, stimulation intensity (mA) was increased after every third consecutive response. Whole-cell patch-clamp recordings of miniature excitatory postsynaptic currents (mEPSCs) were obtained from voltage-clamped (–70 mV) SPNs of the dorsolateral striatum. Recording pipettes (2.5–3.5 MΩ) were filled with an internal solution consisting of 100 mM CsMeSO_4_, 10 mM TEA-Cl, 8 mM NaCl, 10 mM HEPES, 5 mM QX-314-Cl, 2 mM Mg-ATP, 0.3 mM Na-GTP, and 10 mM EGTA (pH 7.25, 295 mOsm). Responses were recorded for 2 min after maintaining a stable baseline for 5 min. Picrotoxin (60 μM) and TTX (1 μM) were included in aCSF to block GABA receptors and voltage-gated Na^+^ channels, respectively.

### Transmission Electron Microscopy

Mice were deeply anesthetized with a mixture of ketamine (120 mg/kg) and xylazine (10 mg/kg) and were intracardially perfused with heparinized normal saline, followed by a freshly prepared fixative of 2.5% glutaraldehyde and 1% paraformaldehyde in 0.1 M phosphate buffer (PB, pH 7.4). Whole brains were post-fixed for 2 h and stored in PB overnight at 4°C. A total of 50 μm-thick coronal sections were osmicated with 1% osmium tetroxide, dehydrated in graded alcohols, flat embedded in Durcupan ACM (Fluka), and then cured for 48 h at 60°C. Dorsolateral striatum regions were cut out of the wafers and glued onto the plastic block with cyanoacrylate. Ultrathin sections were cut and mounted on Formvar-coated single-slot grids, stained with uranyl acetate and lead citrate, and examined with an electron microscope (Hitachi H-7500) at 80 kV accelerating voltage. A total of thirty two micrographs were taken per animal representing 837.9 μm^2^ (27.31 μm^2^ × 32) neuropil regions at a 30,000× magnification. The number of postsynaptic densities (PSDs), the proportion of perforated PSDs, the length and thickness of PSDs, and the number of docked vesicles (as defined by direct contact with presynaptic plasma membrane) per active zone length were quantified by an experimenter blind to the genotype. Digital images were captured with the GATAN DigitalMicrograph software driving a CCD camera (SC1000 Orius, Gatan). The brightness and contrast of the images were adjusted in Adobe Photoshop 7 (Adobe Systems).

### Golgi-Cox Staining

Golgi-Cox staining was conducted following the manufacturer’s protocol of the FD Rapid GolgiStain™ Kit (FD NeuroTechnologies, Inc.). One hundred-micrometer-thick sections were collected using a vibratome (Leica VT 1200S). Wholly stained neurons were selected from the dorsolateral striatum and layer V of the motor cortex of *Dlg2^–/–^* and WT littermate controls using a brightfield microscope (Leica DMi8). SPNs in the dorsolateral striatum were identified based on their morphological features as previously described (Bentea et al., 2020), e.g., either round or ovoid soma with a diameter ranging from 10 to 20μm that contains at least three primary dendrites having a relatively low density of spines that increases as the branching order becomes higher. Layer V pyramidal neurons in the motor cortex were identified as previously described (Urrego et al., 2015) based on morphological features, e.g., a triangular-shaped and relatively large soma that has the apical dendrites projecting toward the pial surface. Entire neurons within an imaging depth of stained sections (approximately 60 μm), excluding axons, were traced at 40× magnification. Note that long dendrites of pyramidal neurons were often partially reconstructed due to cut by sectioning. A total of 8–12 SPNs and 13–15 pyramidal neurons per animal were traced. The degree of dendritic arborization was assessed using the Sholl analysis in that a series of concentric circles (separated by 10 μm in radius) was overlaid over neuron traces and the number of intersections was counted using ImageJ (Schindelin et al., 2012). The spine density was estimated from dendrites greater or equal to the third order for SPNs and to the second order for pyramidal neurons and at least 30 μm distant from cell soma. For spine quantification, SPNs and pyramidal neurons were imaged at 63× magnification. The spine density was assessed on 8–14 neurons per animal. Statistical comparisons were made based on the representative value of each animal that was averaged from sampled neurons per animal. An experimenter was blinded to the group information throughout the data collecting and analysis processes. The brightness and contrast of the images were adjusted in Adobe Photoshop 7 (Adobe Systems).

### Immunoblotting Analysis

For postsynaptic composition analyses, striatum and cortex from 3- to 5-month-old mice were dissected and homogenized with ice-cold buffer (0.32 M sucrose, 10 mM HEPES, pH 7.4, 2 mM EDTA, pH 8, 2 mM EGTA, pH 8, protease inhibitors, phosphatase inhibitors). Synapse enriched fraction was collected as previously described (Chung et al., 2019). A synaptic or crude membrane (P2) fraction lysates separated in electrophoresis and transferred to a nitrocellulose membrane were incubated with primary antibodies to DLG2/PSD-93 (#1634, rabbit), DLG4/PSD-95 (Neuromab 75-028), pan-Shank (Neuromab 75-089), Homer1 (#1133, rabbit), GluA1 (#1193, rabbit), GluA2 (Millipore MAB397), and α-tubulin (Sigma T5168) at 4°C overnight. Fluorescent secondary antibody signals were detected using the Odyssey ^®^ Fc Dual-Mode Imaging System. For the DRD1, DRD2, and TrkA, expression analysis, striatum and/or cortex lysates were separated in electrophoresis and then transferred to a polyvinyldene fluoride (PVDF) membrane, followed by the incubation with anti-TrkA (#2505S, Cell Signaling Technology), anti-DRD1 (#D2944, Sigma Aldrich), anti-DRD2 (#AB5084P, Merck Millipore), anti–GAPDH (#LF-PA0212, ABfrontier) and anti-β-actin (#sc-47778 Santa Cruz Biotechnology) at 4°C overnight and then with secondary antibodies at room temperature for 1 h. Protein bands were visualized using the FujiFilm LAS-4000 Mini Luminescent Image Analyzer.

### Statistical Analysis

Statistical analyses were performed using GraphPad Prism 7. Repeated measures ANOVA was used for analyzing input/output and paired-pulse ratio. *Post hoc* analyses were performed using Sidak’s test, as warranted by significant interaction effects. mEPSCs were analyzed using either Student’s *t*-test for normally distributed data, as determined by the D’Agostino and Pearson’s tests, or by the non-parametric Mann-Whitney test for non-normally distributed data. Student’s *t*-test was used for the analysis of electron microscopy, Golgi-Cox staining, and immunoblotting. All statistical details, including information on sample sizes, descriptive statistics, normality test results, and t-, F-, U- values, are summarized in Supplementary Table 1. Results are presented as means ± SE, and differences with a *p*-value < 0.05 were considered statistically significant.

## Results

### Corticostriatal Synaptic Transmission Is Suppressed in *Dlg2^–/–^* Mice

**FIGURE 1:**
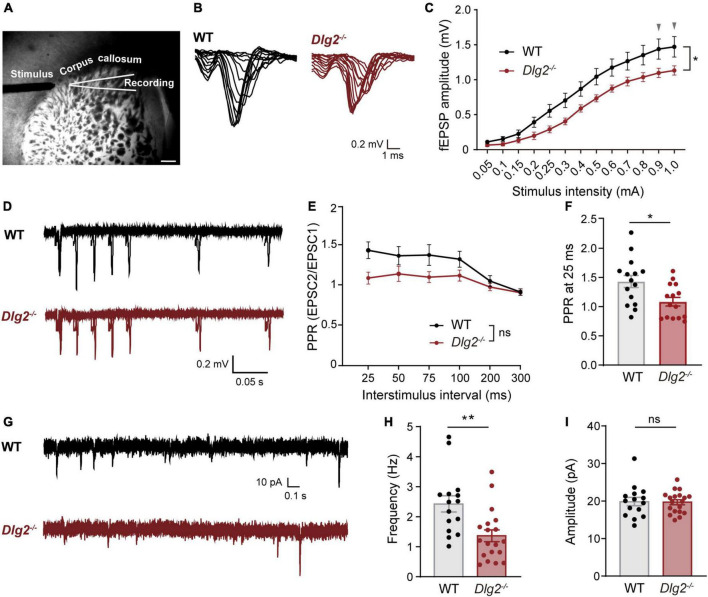
Corticostriatal synaptic transmission is suppressed in *Dlg2^–/–^* mice. **(A)** Representative micrograph of the dorsolateral striatum region showing placement of extracellular field recordings and stimulus *via* the corpus callosum. Scale bar, 400 μm. **(B)** Representative traces of input-output measurements were performed by extracellular field recordings for WT and *Dlg2^–/–^* mice. **(C)** The amplitude of corticostriatal fEPSPs was significantly reduced in *Dlg2^–/–^* mice (*n* = 15 slices from three WT mice and *n* = 15 slices from three *Dlg2^–/–^* mice). Arrowheads indicate *p*-values being 0.05 in *post hoc* Sidak’s multiple comparisons tests (see details for Supplementary Table 1). **(D)** Representative traces of paired-pulse ratio (PPR) from WT and *Dlg2^–/–^* mice. Paired-pulse ratio, as shown by fEPSP amplitude plotted against interstimulus intervals, was not significantly altered overall in *Dlg2^–/–^* mice in repeated measures of ANOVA **(E)**, but at 25 ms **(F)**, it was significantly reduced in *Dlg2^–/–^* mice (*n* = 15 slices from three WT mice and *n* = 15 slices from three *Dlg2^–/–^* mice). **(G)** Representative traces of whole-cell patch-clamp recordings of miniature excitatory postsynaptic currents (mEPSCs) from WT and *Dlg2^–/–^* mice. **(H)** The frequency of mEPSCs was decreased in striatal SPNs of *Dlg2^–/–^* mice compared with that in WT mice, suggesting the corticostriatal dysfunction attributable to a smaller number of functional synapses in the striatum of *Dlg2^–/–^* mice than that of WT. **(I)** There was no group difference in the amplitude of mEPSCs. *n* = 15 neurons from three WT mice and *n* = 19 neurons from four *Dlg2^–/–^* mice. Student’s *t*-test (mEPSC frequency) and the Mann-Whitney test (mEPSC amplitude) were used according to the normality of the data. *p < 0.05; **p < 0.01; ns, not significant.

Corticostriatal circuitries play important roles in the regulation of cognitive and motor function (Shepherd, 2013), including locomotor response to novelty (Ishii et al., 2016; Leonzino et al., 2019), social interactions (Li and Pozzo-Miller, 2020), and repetitive “stereotype” behaviors (Welch et al., 2007; Shmelkov et al., 2010; Peça et al., 2011), all of which are found to be abnormal in *Dlg2^–/–^* mice (Yoo et al., 2020). To investigate whether a DLG2/PSD-93 deficiency causes dysfunction of corticostriatal projections, we measured extracellular field excitatory postsynaptic potentials (fEPSPs) in the dorsolateral striatum evoked by stimulating the corpus callosum, which mainly contains corticostriatal tracts (Figure 1A), as previously described (Peça et al., 2011; Bentea et al., 2020). Compared with WT animals, *Dlg2^–/–^* mice showed significantly decreased fEPSPs in the dorsolateral striatum, with two curves progressively diverging as the stimulation intensity increased (Figures 1B,C), indicating suppressed corticostriatal synaptic transmission in *Dlg2^–/–^* mice. The presynaptic function was not impaired by a DLG2/PSD-93 deficiency, as evidenced by the absence of the main effect of genotypes in paired-pulse ratios (PPRs, Figures 1D,E). Instead, there was a tendency toward an increased probability of presynaptic releases in *Dlg2^–/–^* mice, as shown by the significantly reduced value of PPRs at 25 ms (Figure 1F).

### A Discs’ Large Homologs2/Postsynaptic Density Protein-93 Deficiency Results in the Reduced Number of Functional Excitatory Synapses on Spiny Projection Neurons of the Dorsolateral Striatum

To investigate the physiological driver of the reduction in fEPSPs in the *Dlg2^–/–^* striatum, we performed whole-cell voltage-clamp recordings of mEPSCs in SPNs, one of the major output cell types in the dorsolateral striatum. We found that the frequency, but not amplitude, of mEPSCs in SPNs was significantly decreased in *Dlg2^–/–^* mice (Figures 1G–I), indicating the absence of changes in the strength of individual excitatory synapses (Figure 1I). Given no evidence of impaired presynaptic function (Figures 1D–F), this result indicates that the number of functional synapses is decreased in *Dlg2^–/–^* SPNs.

**FIGURE 2:**
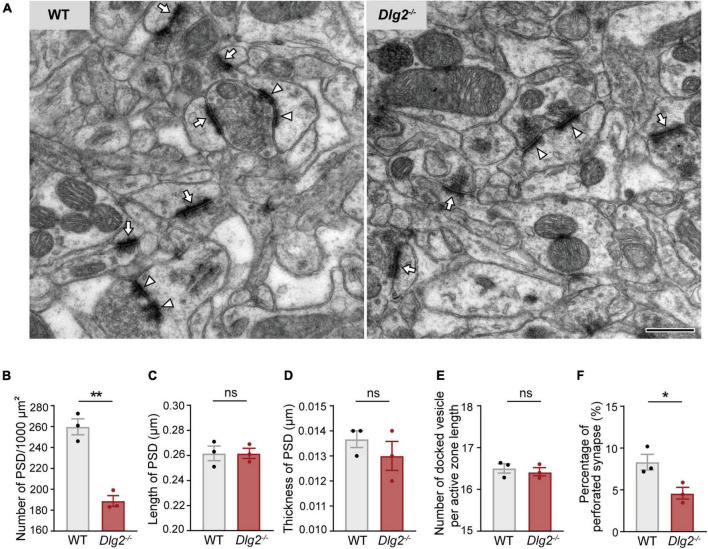
The number of functional synapses is decreased in the dorsolateral striatum of *Dlg2^–/–^* mice. **(A)** Representative electron microscopy images of the dorsolateral striatum of WT and *Dlg2^–/–^* mice. Arrows, synapse; arrowheads, perforated synapse. Scale bar, 500 nm. **(B)** The number of PSDs in the dorsolateral striatum was significantly reduced in *Dlg2^–/–^* mice, corroborating the decreased number of synapses in the dorsolateral striatum. **(C–E)** There were no group differences in the length or thickness of PSDs or the number of presynaptic docked vesicles per active zone length. **(F)** The percentage of perforated synapses was significantly reduced in *Dlg2^–/–^* mice. A total of 32 micrographs per animal were analyzed (WT, *n* = 3 mice; *Dlg2*^–/–^, *n* = 3 mice). *p < 0.05; **p < 0.01; ns, not significant. Student’s *t*-test.

To determine whether the decreased number of functional synapses on *Dlg2^–/–^* SPNs is attributed to the reduction in the total number of excitatory synapses caused by a DLG2/PSD-93 deficiency, quantification of synapses was performed at the ultrastructural level using transmission electron microscopy. The number of PSDs in the dorsolateral striatum was significantly decreased in *Dlg2^–/–^* mice compared with that in wild-type (WT) animals (Figures 2A,B). The percentage of perforated synapses, a type of synapse often regarded as a functionally mature form of axospinous synapses (Geinisman, 1993), was also significantly reduced in *Dlg2^–/–^* mice (Figure 2F). The number of presynaptic docked vesicles per active zone length, as well as the length and thickness of PSDs, were not significantly different between genotypes (Figures 2C–E). Taken together, these findings suggest that a DLG2/PSD-93 deficiency impairs the corticostriatal synaptic transmission, mainly owing to a reduction in the number of functional excitatory synapses in SPNs of the dorsolateral striatum.

### Dendritic Spines of Spiny Projection Neurons in the Dorsolateral Striatum, but Not of Pyramidal Neurons in the Cortex, Were Decreased in *Dlg2^–/–^* Mice

**FIGURE 3:**
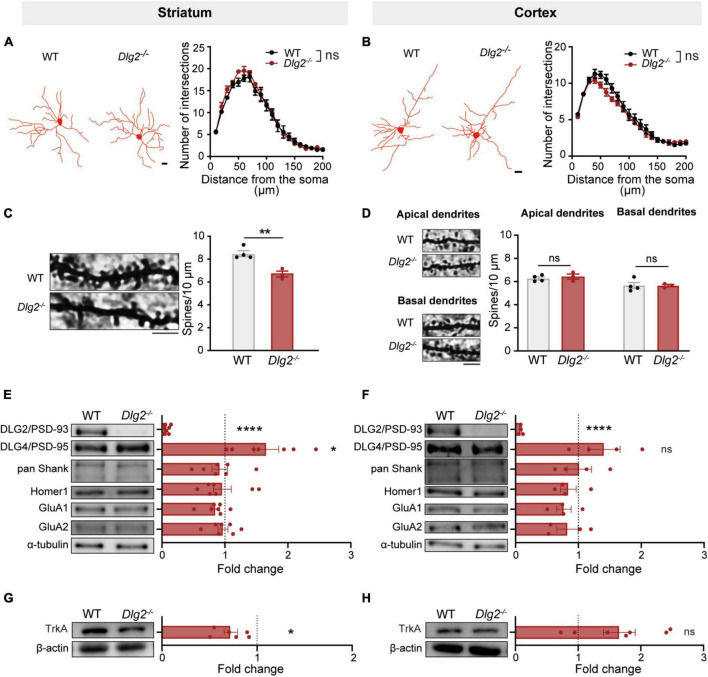
The consequence of a DLG2/PSD-93 deficiency on excitatory synapses is prominent in the striatum compared to that in the motor cortex. **(A,B)** Golgi-Cox staining revealed no changes in dendritic arborization of SPNs in the dorsolateral striatum **(A)** or pyramidal cells in the cortex **(B)** of *Dlg2^–/–^* mice. Left, representative traces. Scale bars, 15 μm; right, quantification by the Sholl analysis. A total of 8–12 SPNs and 13–15 pyramidal neurons per animal were analyzed (WT, *n* = 4 mice; *Dlg2^–/–^*, *n* = 3 mice). **(C,D)** The number of dendritic spines was significantly reduced in striatal *Dlg2^–/–^* SPNs **(C)**, but not in cortical pyramidal neurons **(D)**. Left, representative images of dendrites. Scale bar, 5 μm. A total of 12–15 neurons per animal were analyzed for spine quantification (WT, *n* = 4 mice; *Dlg2^–/–^*, *n* = 3 mice; **p < 0.01, Student’s *t*-test). Representative blot images of postsynaptic density-related molecule in the synapse-enriched fraction of the striatum **(E)** and the cortex **(F)**. Immunoblot analysis confirmed the absence of DLG2/PSD-93 at striatal and cortical synapses in *Dlg2^–/–^* mice. Note that DLG4/PSD-95 protein levels were significantly increased in the striatal, but not the cortical, synaptic fraction of *Dlg2^–/–^* mice. No other changes in synaptic composition were found. **(G,H)** TrkA was significantly decreased in the striatum of *Dlg2^–/–^* mice, but not in the cortex. *p < 0.05; ****p < 0.0001; ns, not significant. Student’s *t*-test.

Given the important role of SPNs in numerous complex striatum-related behaviors (Welch et al., 2007; Shmelkov et al., 2010; Ahmari et al., 2013; Rothwell et al., 2014; Calabresi et al., 2016; Koch et al., 2018), we investigate whether a DLG2/PSD-93 deficiency specifically alters morphological and structural features of SPNs in comparisons with its effect on pyramidal neurons in layer V of the cortex. Golgi-Cox staining results revealed that a DLG2/PSD-93 deficiency did not alter the complexity of dendritic arborization of SPNs in the dorsolateral striatum, nor of pyramidal neurons in the motor cortex (Figures 3A,B). However, the number of dendritic spines of SPNs in the dorsolateral striatum of *Dlg2^–/–^* mice was significantly reduced, compared with that in WT animals (Figure 3C). On the other hand, there were no group differences in the number of dendritic spines on either apical or basal dendrites of pyramidal neurons in the motor cortex (Figure 3D), highlighting that the consequence of a DLG2/PSD-93 deficiency on dendritic spines was more prominent in the striatal SPNs than the cortical pyramidal neurons.

### Alterations in Synapse-Related Molecules Caused by a Discs’ Large Homologs2 Deficiency Are Pronounced in the Striatum, Compared to the Cortex

With the prominent decreases in the number of spines on SPNs in the striatum of *Dlg2^–/–^* mice, we then addressed the question of whether a DLG2/PSD-93 deficiency distinctively alters the composition of excitatory synapses in different brain regions. The expression levels of postsynaptic proteins were measured using the synapse-enriched fraction of either the striatum or cortex. Notably, DLG4/PSD-95 protein expression levels were significantly increased in the synaptic fraction of the striatum of *Dlg2^–/–^* mice (Figure 3E), indicating a possible compensatory response to the DLG2/PSD-93 deficiency as previously reported in the hippocampus (Winkler et al., 2018). However, the level of DLG4/PSD-95 was not significantly increased in the synaptic fraction of the cortex (Figure 3F). No significant differences were found in other PSD-related molecules, including pan-Shank, Homer, and GluA1 and 2 in either the striatum or cortex (Figures 3E,F). Additionally, there was no significant difference between WT and *Dlg2^–/–^* mice in the expression of dopamine D1 and D2 receptors in the striatum, suggesting that a DLG2/PSD-93 deficiency does not alter the expression of dopamine receptors that are linked to either direct or indirect pathways, respectively (Supplementary Figure 1).

It has been recently reported that DLG2 plays an important role in the nerve growth factor (NGF)-induced differentiation through the regulation of its receptor TrkA expression *via* positive feedback interactions between TrkA and DLG2 in human neuroblastoma cells (Siaw et al., 2020). Therefore, we examined the level of TrkA expression using immunoblotting. The TrkA expression level was significantly decreased in the striatum of *Dlg2^–/–^* mice compared to WT animals (Figure 3G), but there was no group difference in its expression in the cortex (Figure 3H). Together, these results suggest that a DLG2/PSD-93 deficiency prominently alters the number and composition of synapses in the striatum, suggesting striatal dysfunction as a major culprit underlying the suppressed corticostriatal transmission.

## Discussion

Genetic variations in DLG2/PSD-93 have been implicated in various disorders, including schizophrenia (Walsh et al., 2008; Kirov et al., 2012; Fromer et al., 2014), intellectual disability (Reggiani et al., 2017), and ASD (Ruzzo et al., 2019), as well as neurodegenerative disorders, such as AD (Hondius et al., 2016; Lawingco et al., 2021; Prokopenko et al., 2022) and Parkinson’s disease (PD) (Foo et al., 2017; Wu et al., 2018; Zhao et al., 2020). However, the neural substrate for the genetic disruption of DLG2/PSD-93 has not been well understood. Here, we present findings that a DLG2/PSD-93 deficiency causes deficits in excitatory synapses, in particular of SPNs in the striatum, and consequent dysfunction of corticostriatal neurotransmission, highlighting the potential role of DLG2 in brain disorders linked to striatum-related circuitries.

Striatal circuits play a crucial role in the regulation of cognitive and motor functions, including reward-oriented movement, sociability, and stereotyped behaviors (Báez-Mendoza and Schultz, 2013; Shepherd, 2013; Burguiere et al., 2015; Graybiel and Grafton, 2015; Jurado-Parras et al., 2020). Dysfunction in corticostriatal synaptic transmission has been implicated in both psychiatric [e.g., autism (Fuccillo, 2016), obsessive-compulsive disorders (Burguiere et al., 2015), schizophrenia (McCutcheon et al., 2019)] and neurodegenerative disorders [e.g., AD (Anderkova et al., 2017) and PD (Kravitz et al., 2010; Anderkova et al., 2017; Lang et al., 2020)]. The reduced corticostriatal synaptic transmission found in *Dlg2^–/–^* mice appeared to be mainly attributable to the insufficiency of available functional synapses in the dorsolateral striatum. Paired-pulse ratios were significantly reduced at the first interstimulus interval in *Dlg2^–/–^* mice, suggesting even a tendency toward increased presynaptic release probability in the context of a DLG2 deficiency, probably as a compensatory response to the reduction in corticostriatal synaptic transmission. Given that the strength of individual excitatory synapses was not altered in *Dlg2^–/–^* SPNs, as measured by the amplitude of mEPSCs, the reduction in the number of functional synapses in the striatum could fully account for the observed corticostriatal dysfunction in *Dlg2^–/–^* mice. Such an inference is corroborated by the decreased densities of PSDs and dendritic spines in the striatum.

The use of electrical stimulation of corpus callosum encompassing cortical fibers is a well-established approach to investigate corticostriatal projections, as numerously employed in previous studies (Welch et al., 2007; Peça et al., 2011; Assous et al., 2019; Bentea et al., 2020; Hadjas et al., 2020). However, it should be noted that the incoming fibers onto the dorsolateral striatum might contain projections not only from the cortex but also from the thalamus (Alloway et al., 2017). Recent evidence suggests that thalamocortical projections play an important role in the regulation of motor behaviors (Díaz-Hernández et al., 2018). Based on the synaptic deficits found in the striatum of *Dlg2^–/–^* mice, it seems reasonable to postulate that a DLG2/PSD-93 deficiency might alter synaptic transmission projecting from the thalamus as well. Furthermore, given that the cortex, striatum, and thalamus are intricately connected at multi-synaptic levels to form the cortico-basal ganglia-thalamus loop (Foster et al., 2021), the striatal synaptic deficits in *Dlg2^–/–^* mice might alter projections to and/or from the thalamus *via* either direct or indirect pathways. Although we did not find significant differences between groups in the expression of dopamine D1 and D2 receptors in the striatum (Supplementary Figure 1), future studies are necessary to investigate whether a DLG2/PSD-93 deficiency influences the synaptic connectivity of direct and/or indirect striatofugal pathways and subsequently, the cortico-basal ganglia-thalamus network.

It should be noted that this study was focused on the quantification of synapse numbers to confirm the reduction of functional synapse numbers, as hinted by the results of electrophysiological analyses. However, it cannot be ruled out that a DLG2/PSD-93 deficiency might affect various morphological and structural features of individual synapses, and consequently synaptic transmission. The 3D reconstruction of spines and dendrites using serial-section electron microscopy is warranted for detailed assessment regarding the potential effect of a DLG2/PSD-93 deficiency on synaptic morphological features in the striatum.

The consequence of a DLG2/PSD-93 deficiency seems more pronounced in the striatum than in the motor cortex, with respect to the number of dendritic spines. It should be noted that we have quantified the number of spines on pyramidal cells of layer V in the motor cortex since the corticostriatal pathways are mainly projected from cells in layer V of the cortex (McGeorge and Faull, 1989; Shepherd, 2013; Sohur et al., 2014). It has been reported that DLG2/PSD-93 plays an important role in the regulation of developmental synaptic maturation in the mouse visual cortex (Favaro et al., 2018). Therefore, it cannot be ruled out that neurons in different cortical regions and/or layers might be more susceptible to a DLG2/PSD-93 deficiency than those in the motor cortex.

Notably, previous findings have drawn attention to a potential role for DLG2/PSD-93 on striatal development. DLG2/PSD-93 is highly expressed in both the human and mouse striatum during early development (Kang et al., 2011) and its genetic variants are also associated with altered striatal volume in humans (Hibar et al., 2015). We show that a DLG2/PSD-93 deficiency confers the vulnerability to synaptic deficits in the striatum. Although the underlying mechanism remains to be determined, the decreased expression of TrkA in the striatum of *Dlg2^–/–^* mice might imply a clue. TrkA is abundantly expressed in the striatum, as well as the basal forebrain, and it is well known to play an important role in the maturation of cholinergic synapses (Sanchez-Ortiz et al., 2012). A study on high-risk neuroblastomas having a genetic disruption of the *Dlg2* gene has shown that DLG2/PSD-93 expression drives differentiation of neuroblastoma cells *via* increases in the TrkA expression (Siaw et al., 2020). Given that DLG2/PSD-93 is abundantly expressed at neuronal cholinergic synapses (Temburni et al., 2004) and regulates their synaptic stability (Parker et al., 2004), it seems reasonable to postulate that the vulnerability of the striatum to a DLG2/PSD-93 deficiency might be attributed to its role at cholinergic synapses that are abundantly expressed in the striatum. It remains to be determined whether a DLG2/PSD-93 deficiency specifically causes a loss of cholinergic synapses in the striatum and if so, whether it affects other cholinergic systems, such as basal forebrain.

Although our previous study has showed that DLG4/PSD-95 is not altered in the whole brain of *Dlg2^–/–^* mice (Yoo et al., 2020), the present study revealed that its expression is significantly increased in the crude synaptic fraction of the *Dlg2^–/–^* striatum. This finding is reminiscent of the results of a previous study showing that DLG2/PSD-93 is significantly increased in the hippocampal synaptic fraction of *Dlg4*/PSD-95^+/–^ mice (Winkler et al., 2018). Given the role of these DLG family members in the regulation of AMPA and NMDA receptors in PSDs (Chen et al., 2015), it is possible that the increased expression of DLG4/PSD-95 in the striatal synaptic fraction might, in part, account for the absence of changes in the postsynaptic efficacy of *Dlg2^–/–^* SPNs. Future studies should determine how synapses in different brain regions and circuitries cope with a DLG2/PSD-93 deficiency at PSDs and what the functional ramifications of overexpression of DLG4/PSD-95 might be on the organization of postsynaptic receptors and other interacting molecules, including Shank (Naisbitt et al., 1999).

DLG2/PSD-93 has been largely viewed as merely one of the MAGUK family members with *apparent* biological redundancy with DLG4/PSD-95. We previously identified an important role for DLG2/PSD-93 in behavioral abnormalities, including a reduction in social interaction, abnormal locomotor responses to novelty, and heightened repetitive behavior. The results presented here indicate that genetic disruption of DLG2/PSD-93 leads to a reduction in the number of spines and functional excitatory synapses on SPNs in the dorsolateral striatum, with attendant dysfunction of corticostriatal synaptic transmission. Given that corticostriatal circuitry is strongly implicated in cognitive and motor function, these findings provide clues that may help unveil the pathological role of DLG2/PSD-93 in psychiatric and neurological disorders involving dysregulation of striatal circuits.

## Data Availability Statement

The raw data supporting the conclusions of this article will be made available by the authors, without undue reservation.

## Ethics Statement

The animal study was reviewed and approved by the committees’ of animal research at KAIST (KA2021-070) and Yeungnam University (2020-037).

## Author Contributions

TY and SK conceived the project and designed experiments. TY performed electrophysiological recordings. SJ performed Golgi-Cox staining and immunohistochemistry. TY, SJ, SP, and JK performed western blotting. YC performed electron microscopy analysis under YB’s supervision. TY, SJ, SP, YC, P-HP, and SK analyzed the data and interpreted results. TY, SJ, and SK wrote and edited the manuscript. EK and SK supervised the study. All authors read and approved the final manuscript.

## Conflict of Interest

The authors declare that the research was conducted in the absence of any commercial or financial relationships that could be construed as a potential conflict of interest.

## Publisher’s Note

All claims expressed in this article are solely those of the authors and do not necessarily represent those of their affiliated organizations, or those of the publisher, the editors and the reviewers. Any product that may be evaluated in this article, or claim that may be made by its manufacturer, is not guaranteed or endorsed by the publisher.

## Supplementary Material

Supplementary Figure 1 The expression of dopamine D1 and D2 receptors is not altered in the striatum of *Dlg2^–/–^* mice. **(A)** Representative images of immunohistochemical analysis for the expression of dopamine D1 and D2 receptors (DRD1 and DRD2, respectively) in the striatum of WT and *Dlg2^–/–^* mice. Scale bar, 50 μm. **(B)** There was no significant difference in the optical densities of DRD1 and DRD2 in the striatum between groups. A total of 3 sections were used per animal (WT, *n* = 4, *Dlg2^–/–^* mice, *n* = 4). **(C)** Representative immunoblot images of the DRD1 and DRD2 expression in the lysates of the striatum. **(D)** The expression levels of DRD1 and DRD2 were not altered by a DLG2/PSD-93 deficiency in the striatum. Note that all three bands in DRD2 were included for quantification. WT, *n* = 6, *Dlg2^–/–^* mice, *n* = 6. ns, not significant; Student’s *t*-test.
