# Supplementary material for: A Deficiency of the Psychiatric Risk Gene DLG2/PSD-93 Causes Excitatory Synaptic Deficits in the Dorsolateral Striatum
Source: Front Mol Neurosci. 2022 Jul 28;15:938590. doi: 10.3389/fnmol.2022.938590 (PMC9370999; doi:10.3389/fnmol.2022.938590)
Supplement: Supplementary file 1 [file Data_Sheet_1.pdf]

Supplementary methods

Immunohistochemistry

Transcardially perfused brains (ice-cold 4% paraformaldehyde in phosphate buffered saline) of 3 months old male mice were sectioned at a thickness of 40 μm using vibratome (Leica VT 1200S). Free-floating immunohistochemistry was performed as previously described (Yook et al., 2019, Frontiers in Molecular Neuroscience 12:241). In brief, the brain sections were permeabilized with TBS buffer containing 0.3% Triton-X and then blocked with 2% normal donkey serum (#017-000-121, Jackson ImmunoResearch) at room temperature for 30 minutes. Sections were then incubated overnight with primary antibodies for DRD1 (diluted at 1:200) and DRD2 (1:400) at 4°C, followed by a series of washes and incubation with secondary antibodies at room temperature for two hours. Using a brightfield microscope (Leica DMi8), images were captured at the 40X magnification from the dorsolateral striatum. The optical density was measured using ImageJ. Three sections per animal (WT, n = 4, *Dlg2*$^{-/-}$, n = 4) were used for quantification.
