# Supplementary figures and images for: A Deficiency of the Psychiatric Risk Gene DLG2/PSD-93 Causes Excitatory Synaptic Deficits in the Dorsolateral Striatum
Source: Front Mol Neurosci. 2022 Jul 28;15:938590. doi: 10.3389/fnmol.2022.938590 (PMC9370999; doi:10.3389/fnmol.2022.938590)

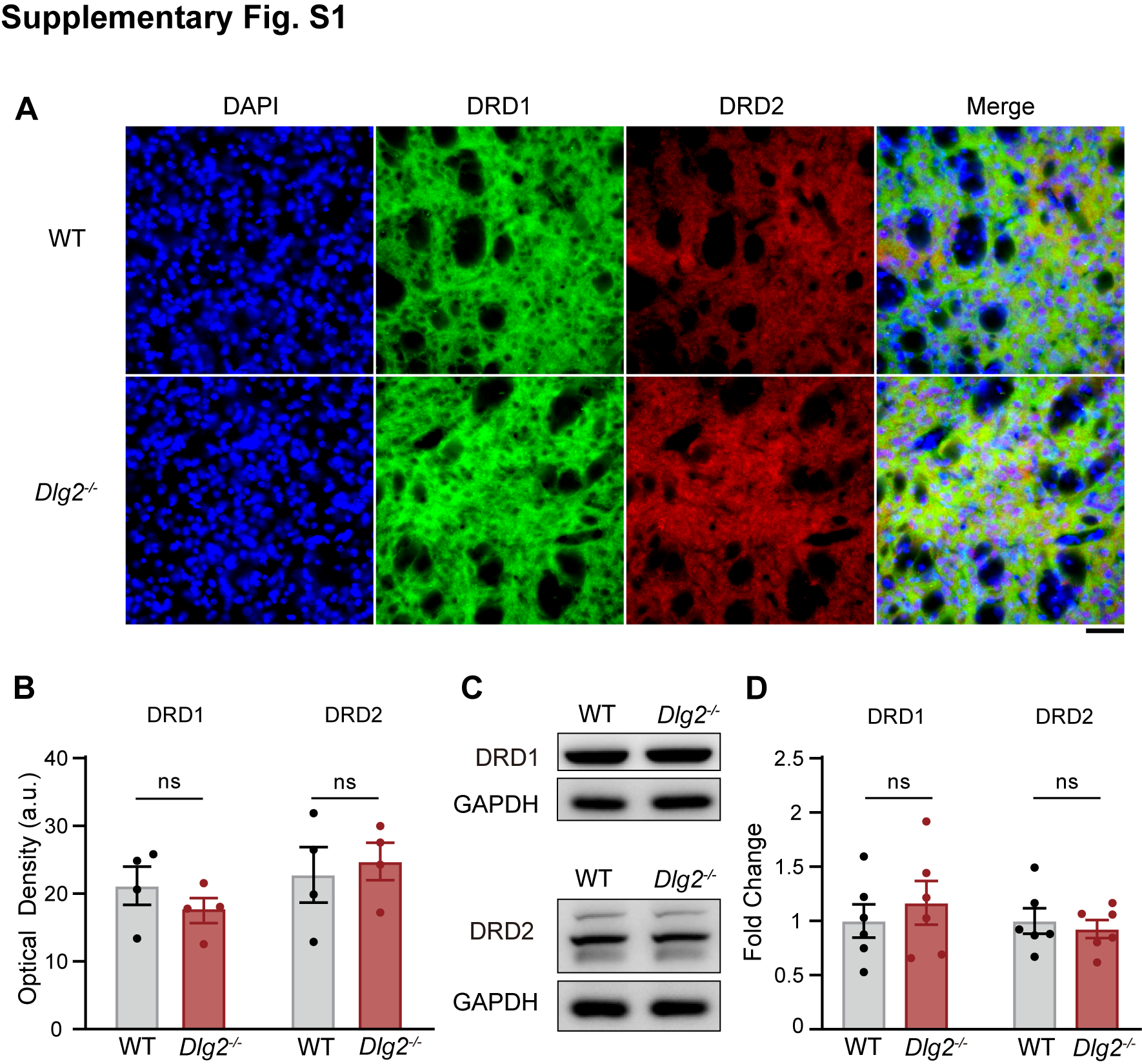

Supplement: Supplementary Figure 1 — The expression of dopamine D1 and D2 receptors is not altered in the striatum of Dlg2–/– mice. (A) Representative images of immunohistochemical analysis for the expression of dopamine D1 and D2 receptors (DRD1 and DRD2, respectively) in the striatum of WT and Dlg2–/– mice. Scale bar, 50 μm. (B) There was no significant difference in the optical densities of DRD1 and DRD2 in the striatum between groups. A total of 3 sections were used per animal (WT, n = 4, Dlg2–/– mice, n = 4). (C) Representative immunoblot images of the DRD1 and DRD2 expression in the lysates of the striatum. (D) The expression levels of DRD1 and DRD2 were not altered by a DLG2/PSD-93 deficiency in the striatum. Note that all three bands in DRD2 were included for quantification. WT, n = 6, Dlg2–/– mice, n = 6. ns, not significant; Student’s t-test. [file Image_1.TIF]
